# Neuroprotective effect of Ruminococcus albus on oxidatively stressed SH-SY5Y cells and animals

**DOI:** 10.1038/s41598-017-15163-5

**Published:** 2017-11-06

**Authors:** Jieun Park, Jiyun Lee, Zia Yeom, Donghyuk Heo, Young-Hee Lim

**Affiliations:** 1 Department of Public Health Science (Brain Korea 21 PLUS program), Graduate School, Korea University, Seoul, 136–701 Republic of Korea; 2 School of Biosystem and Biomedical Science, College of Health Science, Korea University, Seoul, 136–701 Republic of Korea; 3 Department of Laboratory Medicine, Korea University Guro Hospital, Seoul, 152–703 Republic of Korea

## Abstract

Recent evidence shows that the gut microbiota has an important role in gut-brain crosstalk and is linked to neuronal disorders. The aim of this study was to investigate the effects of intestinal *Ruminococcus albus* with probiotic potential on neuroprotection in oxidatively stressed SH-SY5Y neuroblastoma cells and animals. To investigate these effects, conditioned medium was prepared using Caco-2 cells cultured with heat-killed *R*. *albus* (CRA-CM). Caco-2 cells cultured with heat-killed *R*. *albus* showed increased *BDNF* expression and BDNF protein levels increased in CRA-CM. CRA-CM up-regulated the protein expression levels of SRF, C-fos and CDK2. In addition, CRA-CM protected SH-SY5Y cells from H_2_O_2_-induced cell death. CRA-CM significantly decreased the Bax/Bcl-2 ratio in oxidatively stressed SH-SY5Y cells. Animal experiments showed that oral administration of heat-killed *R*. *albus* for 15 days attenuated the oxidative stress induced by sodium arsenate. Treatment with heat-killed *R*. *albus* reduced the level of ROS, and the levels of SOD and GSH increased in oxidatively stressed brains. In conclusion, the secretome prepared from Caco-2 cells cultured with heat-killed *R*. *albus* might promote neuronal proliferation through the activation of cell proliferation-related proteins, and heat-killed *R*. *albus* protects neurons from oxidative damage by reducing ROS levels and increasing SOD and GSH levels.

## Introduction

Mounting evidence suggests that the main cause of neurodegenerative diseases is the misfolding of proteins and dysfunction of the ubiquitin pathway^1^, and oxidative stress and mitochondrial dysfunction may cause an accumulation of misfolded proteins. It is widely accepted that oxidative stress and cytotoxicity of reactive oxygen species (ROS) can cause cell death of nigrostriatal dopaminergic neurons in Parkinson’s disease (PD)^2–4^, indicating that ROS and oxidative stress play an important role in PD. Therefore, numerous studies have focused on attenuating oxidative stress and ROS cytotoxicity to treat PD^5–8^. However, the mechanisms of PD are complicated and remain to be fully elucidated^9^. ROS, which are natural products of cellular processes, can be produced from endogenous and exogenous sources in many ways. ROS are essential for cellular function and can be metabolized safely by antioxidant mechanisms; however, excessive ROS production causes oxidative stress^10^. Excessive ROS can stimulate free-radical chain reactions, which can damage lipids, proteins and DNA and ultimately cause adverse health effects^11^ such as cardiovascular disease, neurodegenerative disorders, ageing, diabetes, cancer and metabolic syndromes^12^. In particular, the brain is known as one of the critical organs susceptible to the damaging effects of ROS. Therefore, antioxidants are promising agents to treat various ROS-associated diseases such as cardiovascular disease, diabetes and neurodegenerative disorders^13,14^.

Recent neurobiological insights into gut-brain crosstalk have revealed a bidirectional communication system that not only ensures the maintenance of gastrointestinal homeostasis and digestion but is also likely to have multiple effects on the brain, including motivation and higher cognitive functions^15^. The gut microbiota has an important role in gut-brain crosstalk and is also linked to neuropsychological disorders^16^. Gut microbiota may be modulated using probiotics, antibiotics and faecal microbiota transplantations, which suggests the possibility of therapy using probiotics and gut microbiota to treat microbiota-associated diseases^17^. It has been assumed that probiotic bacteria need to be alive to confer health benefits on the body when administered in an adequate amount; however, there have been concerns that live bacteria could cause unwanted side effects. To avoid these unwanted side effects, heat- or UV-inactivated bacteria have been assessed as a substitute, and their beneficial effects on the body were found to be similar to the benefits of live bacteria^6,18,19^. In addition, heat- or UV-inactivated bacteria possess several advantages such as safe, stable and easy handling compared with live bacteria.

*Ruminococcus albus* is one of the cellulolytic bacteria considered to play an important role in fibre breakdown in the rumen^20^. *R*. *albus*, an anaerobic ruminal bacterium, produces acetate, ethanol, formate, hydrogen and carbon dioxide from carbohydrates. Hydrogen-producing *R*. *albus* is more abundant in healthy individuals than in patients with Crohn’s disease^21^ and shows probiotic effects^22^. *R*. *albus* was not found in the stools of children with autism, whereas a significant number of *R*. *albus* was found in the stools of control children^23^. Based on that finding, we hypothesized that gut bacteria, especially a strain that is abundant in healthy individuals, could act through the gut-brain axis to attenuate neurodegenerative disorders without any side effects. To test the hypothesis, we investigated the neuroprotective effect of heat-killed *R*. *albus* on oxidatively stressed SH-SY5Y cells and animals. First, we investigated whether heat-killed *R*. *albus* induces Caco-2 cells to produce any factors that might affect neuronal proliferation. Towards this end, conditioned medium (CRA-CM) was prepared using Caco-2 cells treated with heat-killed *R*. *albus*, and cell proliferation and cell protection against oxidative stress were measured in human neuroblastoma SH-SY5Y cells treated with CRA-CM. Second, heat-killed *R*. *albus* was administered to oxidatively stressed animals, and the neuroprotective effect of heat-killed *R*. *albus* on such animals was investigated. As heat- and UV-inactivated bacteria can influence the body in similar ways, we used heat-killed bacteria to evaluate the effect stably and safely. In the animal study, we used an arsenic acid-induced rat model of oxidative stress to evaluate the effect of heat-killed *R*. *albus* on neuroprotection.

## Results

### Effects of CRA-CM on cell viability in SH-SY5Y

**Figure 1:**
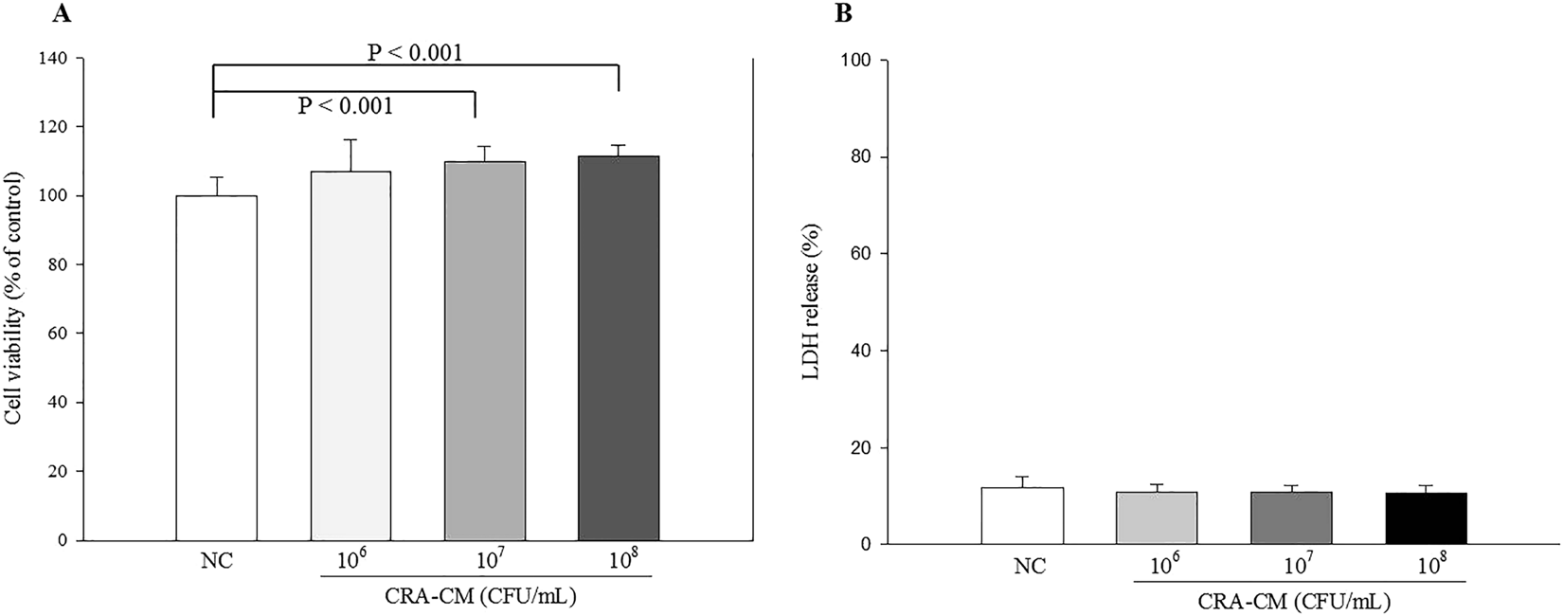
Effects of CRA-CM on cell viability in SH-SY5Y cells. SH-SY5Y cells were treated with CRA-CM for 24 h. Cell viability was measured by MTT (**A**) and LDH (**B**) assay. The data are representative of three independent experiments performed in triplicate.

To evaluate the effect of CRA-CM on cell viability in SH-SY5Y cells, MTT and lactate dehydrogenase (LDH) assays were performed. The viability of CRA-CM-treated SH-SY5Y cells increased in a dose-dependent manner compared with the negative control prepared from Caco-2 cells treated with phosphate-buffered saline (PBS) (Fig. 1). The cell viability measurements were 106.9 ± 9.37%, 110.0 ± 4.55% and 111.5 ± 3.34% in cells treated with CRA-CM prepared with heat-killed *R*. *albus* at concentrations of 10^6^ CFU/mL, 10^7^ CFU/mL and 10^8^ CFU/mL, respectively, compared with the negative control (100%). The cell viability significantly increased in cells treated with CRA-CM prepared with heat-killed *R*. *albus* at concentrations of 10^7^ CFU/mL and 10^8^ CFU/mL compared with the negative control. In the LDH assay, CRA-CM did not show any significant cytotoxicity in SH-SY5Y cells at any of the concentrations used in this study.

### Effects of CRA-CM on cell proliferation-associated factors in SH-SY5Y cells

**Figure 2:**
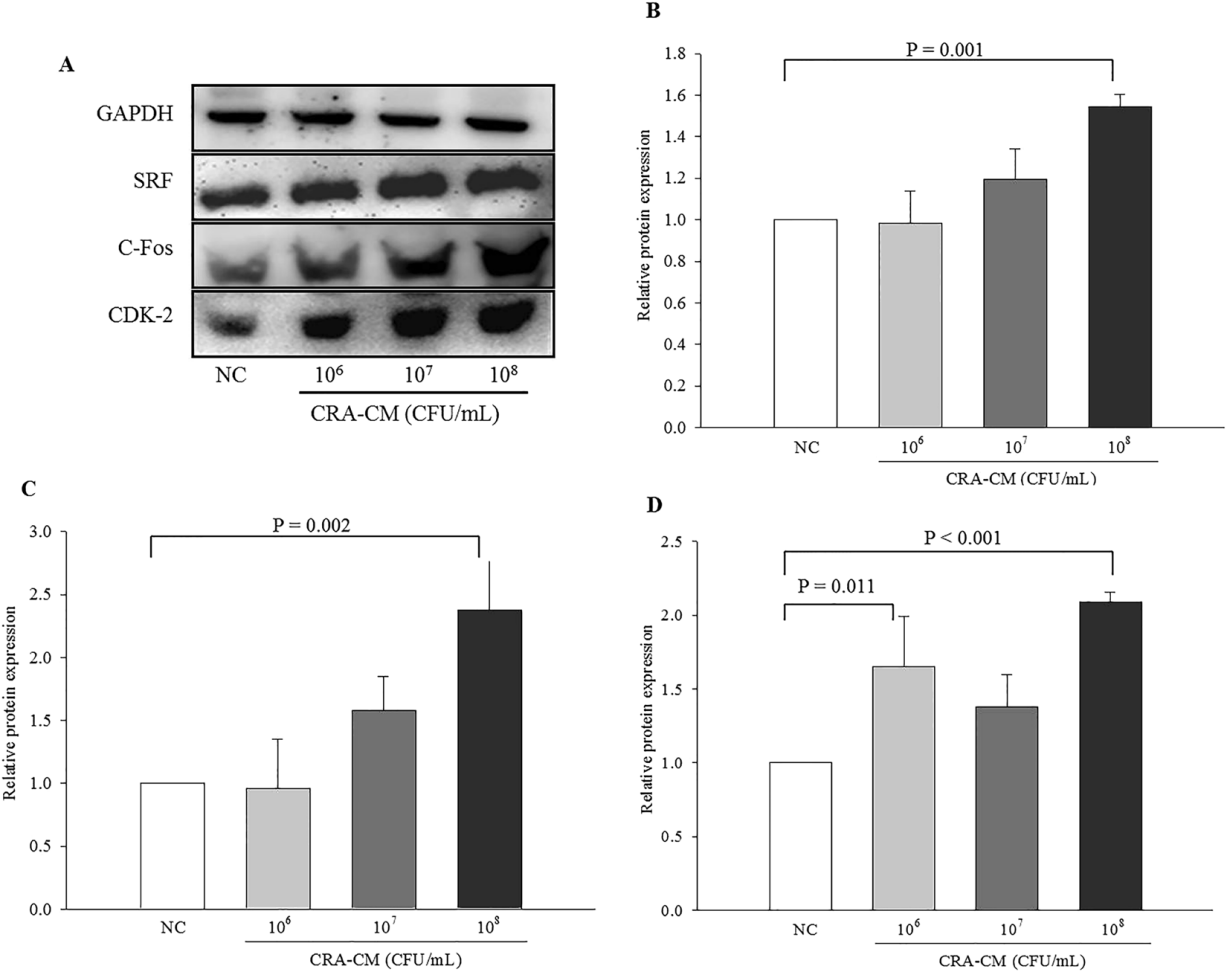
Effects of CRA-CM on the expression of SRF, C-fos and CDK2 in SH-SY5Y cells. The expression levels of SRF, C-fos and CDK2 were measured by western blotting and cropped blots are displayed (**A**), and the densities of SRF (**B**), c-Fos (**C**) and CDK2 (**D**) were quantified. The full-length blots are shown in Supplementary Fig. S2. The data are expressed as the mean ± SD of three independent experiments performed in triplicate.

To investigate the effect of CRA-CM on the expression of cell proliferation-associated factors in SH-SY5Y cells, the expression levels of serum response factor (SRF) and SRF-related proteins were measured by western blot analysis. Expression levels of SRF, C-fos and cyclin-dependent kinase 2 (CDK2) increased in the cells treated with CRA-CM produced with heat-killed *R*. *albus* (Fig. 2A). Expression levels of SRF, a transcription factor that plays an important role in cellular differentiation and cell cycle regulation, increased in the cells treated with CRA-CM that was produced with heat-killed *R*. *albus* at concentrations of 10^6^ CFU/mL (0.98 ± 0.16-fold), 10^7^ CFU/mL (1.19 ± 0.15-fold) and 10^8^ CFU/mL (1.54 ± 0.06-fold) compared with the negative control, especially a significant increase was shown in the cells treated with CRA-CM produced with heat-killed *R*. *albus* at a concentration of 10^8^ CFU/mL (Fig. 2B). C-fos, a known IEG (immediate-early gene), increased in the cells treated with CRA-CM that was produced with heat-killed *R*. *albus* at concentrations of 10^6^ CFU/mL (0.95 ± 0.39-fold), 10^7^ CFU/mL (1.58 ± 0.27-fold) and 10^8^ CFU/mL (2.37 ± 0.41-fold) compared with the negative control, especially a significant increase was shown in the cells treated with CRA-CM produced with heat-killed *R*. *albus* at a concentration of 10^8^ CFU/mL (Fig. 2C). CDK2, also known as cell division protein kinase 2, increased in the cells treated with CRA-CM that was produced with heat-killed *R*. *albus* at concentrations of 10^6^ CFU/mL (1.65 ± 0.34-fold), 10^7^ CFU/mL (1.38 ± 0.22-fold) and 10^8^ CFU/mL (2.09 ± 0.07-fold) compared with the negative control, especially the cells treated with CRA-CM produced with heat-killed *R*. *albus* at concentrations of 10^6^ CFU/mL and 10^8^ CFU/mL significantly increased expression level of CDK2 (Fig. 2D).

### Effects of heat-killed *R*. *albus* on *BDNF* expression in the Caco-2 cell line and BDNF levels in CRA-CM

**Figure 3:**
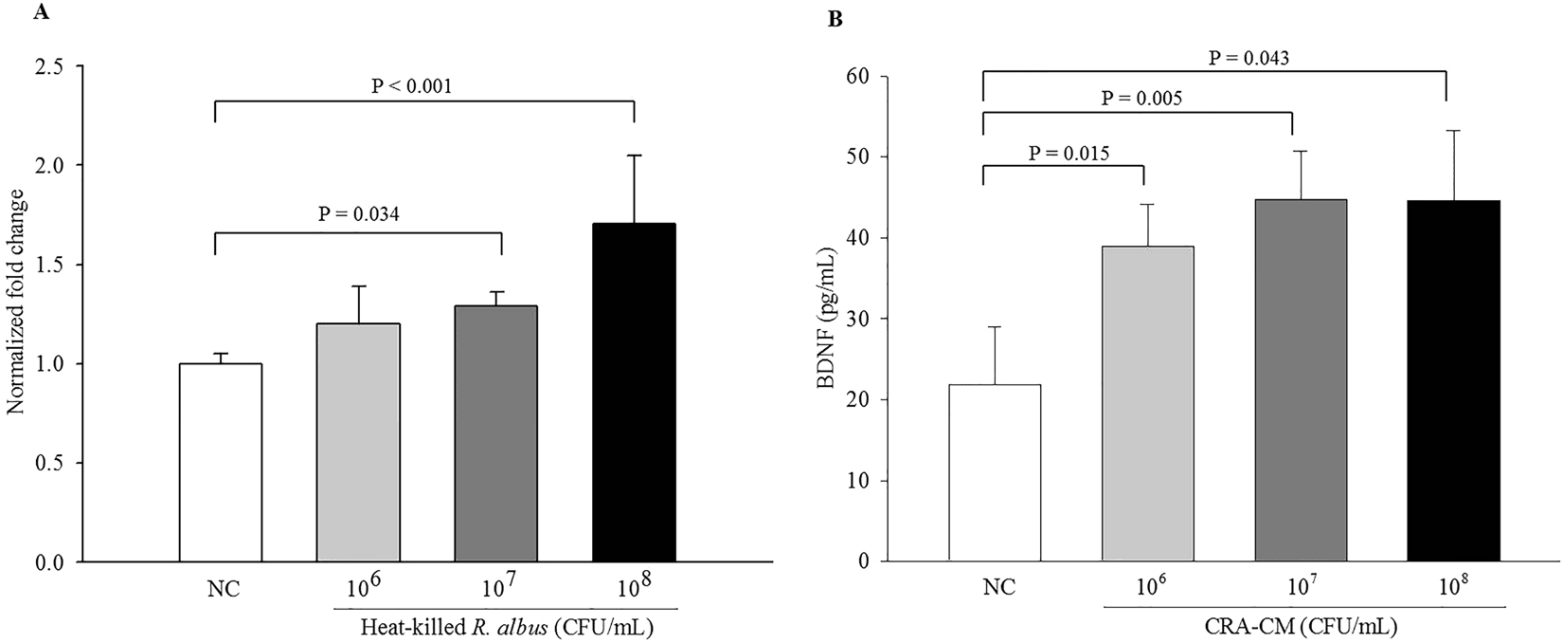
mRNA expression of BDNF in Caco-2 cells treated with heat-killed *R*. *albus* (**A**) and BDNF levels in CRA-CM **(B**). The relative levels of mRNA expression were normalized using *GAPDH* as an internal control. BDNF protein levels in CRA-CM were measured by ELISA. The negative control was treated with PBS. The data are representative of three independent experiments performed in triplicate.

To confirm that Caco-2 cells treated with heat-killed *R*. *albus* express *BDNF* (brain-derived neurotrophic factor) at increased levels, qPCR analysis was performed. Expression levels of *BDNF* in Caco-2 cells treated with heat-killed *R*. *albus* at concentrations of 10^6^ CFU/mL, 10^7^ CFU/mL and 10^8^ CFU/mL increased 1.20-, 1.29- and 1.71-fold, respectively, compared with the negative control (Fig. 3A). BDNF levels in CRA-CM were measured by ELISA. BDNF levels significantly increased in CRA-CM produced with heat-killed *R*. *albus* compared with the negative control (Fig. 3B). BDNF levels in CRA-CM from 10^6^ CFU/mL, 10^7^ CFU/mL and 10^8^ CFU/mL *R*. *albus* increased 1.78-, 2.05- and 2.04-fold, respectively, compared with the negative control.

### Effects of CRA-CM on expression of β-tubulin in SH-SY5Y cells

**Figure 4:**
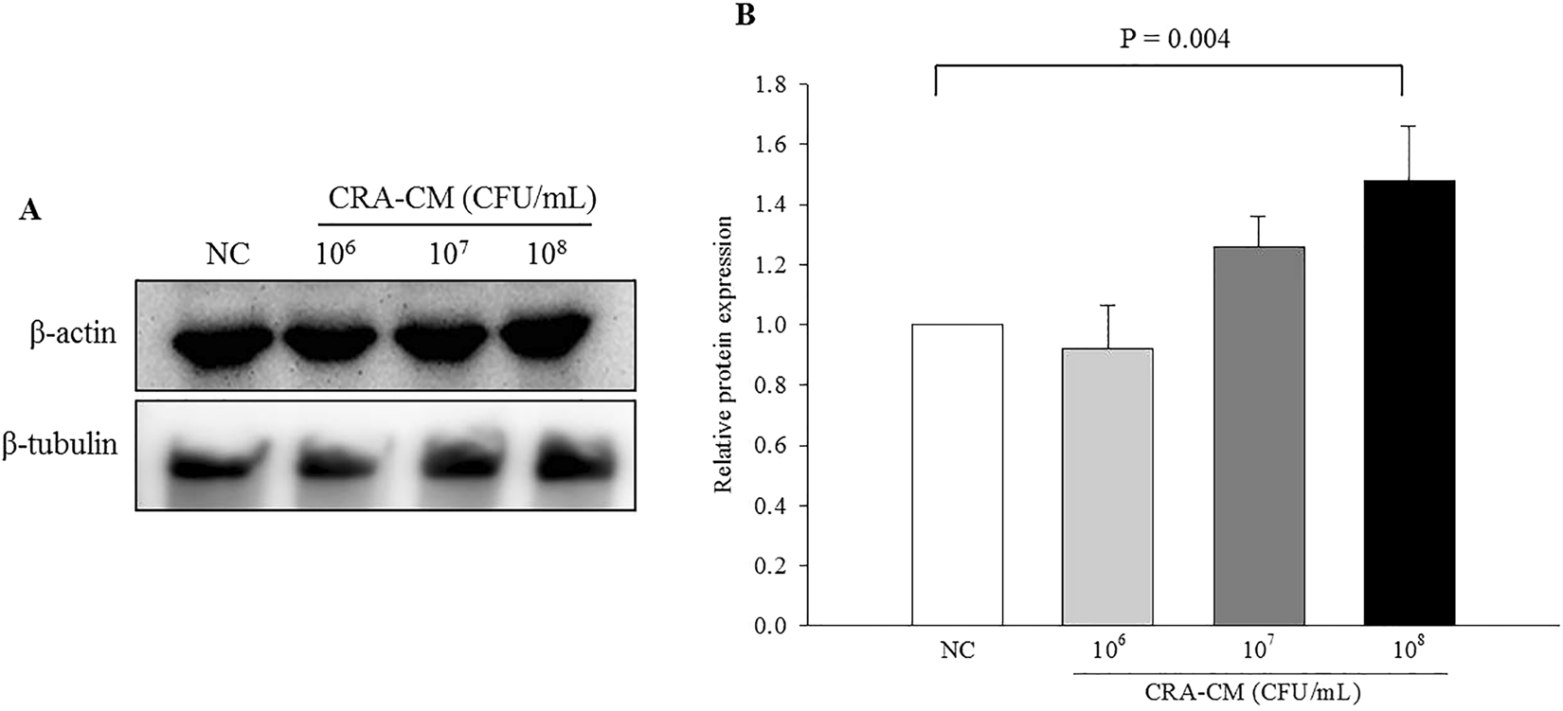
Effects of CRA-CM on expression of β-tubulin in SH-SY5Y cells. Expression levels of β-tubulin were measured by western blotting and cropped blots are displayed (**A**), and quantified (**B**). The full-length blots are shown in Supplementary Fig. S3. The data are expressed as the mean ± SD of three independent experiments performed in triplicate.

Two-dimensional gel analysis was used to compare protein expression levels between CRA-CM-treated and CRA-CM-untreated SH-SY5Y cells. A protein spot was selected by its pronounced difference in expression (354.37-fold increased) compared with the negative control (Supplementary Fig. S1). The protein was identified as β-tubulin, which is found in developing and regenerating neurons. To confirm the increased expression of β-tubulin, western blot analysis was performed (Fig. 4A). In cells treated with CRA-CM from 10^6^ CFU/mL, 10^7^ CFU/mL and 10^8^ CFU/mL *R*. *albus*, the protein expression levels of β-tubulin were 0.92, 1.26 and 1.48 times the negative control value, respectively (Fig. 4B). The protein expression levels of β-tubulin significantly increased in the cells treated with CRA-CM produced with heat-killed *R*. *albus* at a concentration of 10^8^ CFU/mL.

### Neuroprotective effects of CRA-CM on oxidatively stressed SH-SY5Y cells

**Figure 5:**
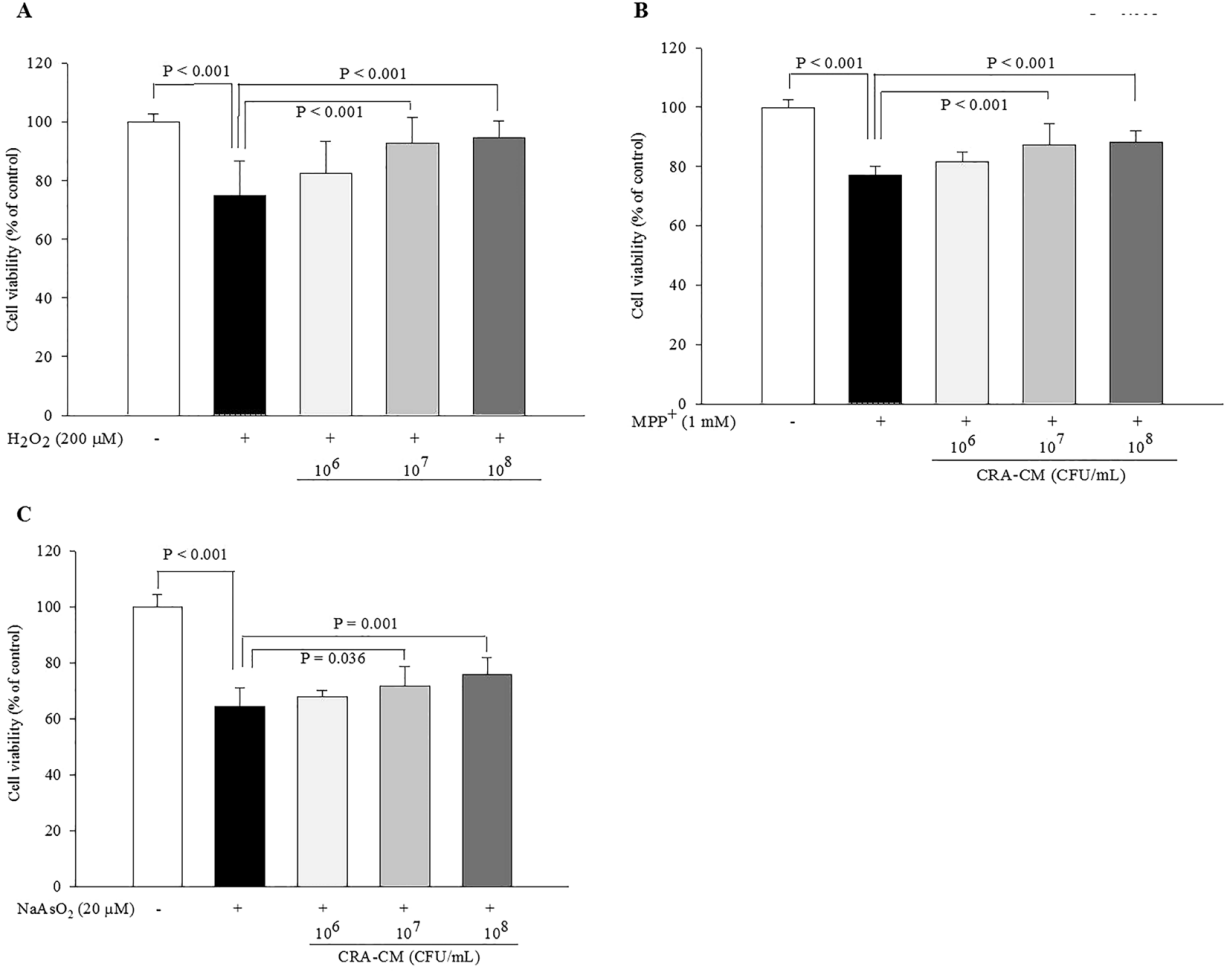
Neuroprotective effects of CRA-CM on cell viability in oxidatively stressed SH**-**SY5Y cells. Oxidative stress was induced by (**A**) H_2_O_2_ (200 μM), (**B**) MPP^+^ (1 mM) or (**C**) NaAsO_2_ (20 μM). SH-SY5Y cells were pretreated with CRA-CM. After 4 h, H_2_O_2_, MPP^+^ or NaAsO_2_ was applied for 20 h. Cell viability was measured using an MTT assay. The data are representative of three independent experiments performed in triplicate.

Cell viability significantly decreased to 74.93 ± 11.68%, 77.19 ± 2.90% and 64.38 ± 6.84% in cells treated with the oxidative stress inducers H_2_O_2_ (200 μM), MPP^+^ (1 mM) and NaAsO_2_ (20 μM), respectively, compared with the negative control (100%). However, the effect of oxidative stress on cell viability was mitigated in a dose-dependent manner by treatment with CRA-CM. The viability of cells oxidatively stressed with H_2_O_2_ (74.93 ± 11.68%) increased to 82.61 ± 10.88%, 92.70 ± 8.97% and 94.76 ± 5.68% under co-treatment with CRA-CM from 10^6^ CFU/mL, 10^7^ CFU/mL and 10^8^ CFU/mL heat-killed *R*. *albus*, respectively. One-way ANOVA analysis showed a significant variation of heat-killed *R*. *albus* (*p* < 0.001, *F*-value = 11.5, *df*_1_ = 4, *df*_2_ = 39) (Fig. 5A). The viability of cells oxidatively stressed with MPP^+^ (77.19 ± 2.90%) increased to 81.65 ± 3.24%, 87.16 ± 7.32% and 88.04 ± 4.00% under co-treatment with CRA-CM from 10^6^ CFU/mL, 10^7^ CFU/mL and 10^8^ CFU/mL heat-killed *R*. *albus*, respectively One-way ANOVA analysis showed a significant variation of heat-killed *R*. *albus* (*p* < 0.001, *F*-value = 33.5, *df*_1_ = 4, *df*_2_ = 40) (Fig. 5B). The viability of cells oxidatively stressed with NaAsO_2_ (64.38 ± 6.84%) increased to 67.82 ± 2.41%, 71.61 ± 7.23% and 75.78 ± 6.25% under co-treatment with CRA-CM from 10^6^ CFU/mL, 10^7^ CFU/mL and 10^8^ CFU/mL heat-killed *R*. *albus*, respectively. One-way ANOVA analysis showed a significant variation of heat-killed *R*. *albus* (*p* < 0.001, *F*-value = 54.6, *df*_1_ = 4, *df*_2_ = 40) (Fig. 5C). These results show that CRA-CM prepared from heat-killed *R*. *albus* at concentrations of 10^7^ CFU/mL and 10^8^ CFU/mL significantly attenuated cytotoxicity in oxidatively stressed SH-SY5Y cells; in particular, H_2_O_2_-induced oxidative stress was greatly attenuated by treatment with CRA-CM prepared from heat-killed *R*. *albus* at a concentration of 10^8^ CFU/mL.

### Anti-apoptotic effects of CRA-CM in oxidatively stressed SH-SY5Y cells

**Figure 6:**
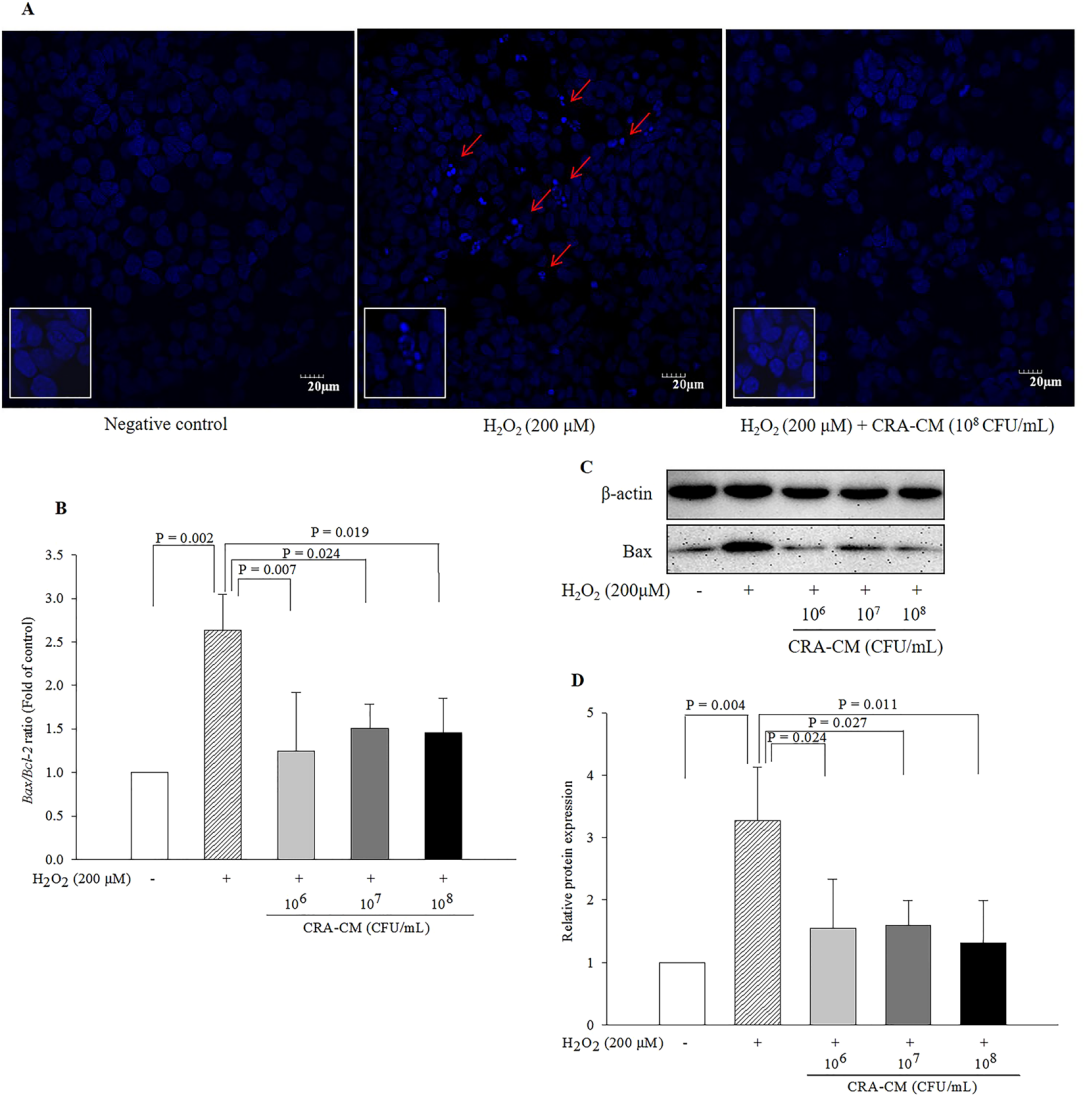
Protective effects of CRA-CM against fragmentation of nuclei in oxidatively stressed SH-SY5Y cells. (**A**) Hoechst 33258 (5 μg/mL) staining was performed in SH-SY5Y cells. Apoptotic cells were identified by the condensation and fragmentation of their nuclei (red arrow). The negative control Caco-2 cells were treated with PBS. The expression levels of apoptosis-related genes and proteins (Bcl-2 and Bax) in oxidatively stressed SH-SY5Y cells were measured by qPCR (**B**) and western blotting and cropped blots are displayed (**C**), respectively. Western blot analysis was quantified using β-actin as an internal control (**D**). The full-length blots are shown in Supplementary Fig. S4. The data are representative of three independent experiments performed in triplicate.

Hoechst 33258 staining was used to detect apoptotic nuclei. The nuclei of normal cells were stained homogenously, whereas the stained nuclei of apoptotic cells appeared shrunk and fragmented. SH-SY5Y cells were pretreated with CRA-CM for 4 h and then treated with H_2_O_2_ (200 μM) for 12 h. The number of apoptotic cells increased under H_2_O_2_ treatment, and the number of shrunk and fragmented nuclei increased in cells treated with H_2_O_2_ alone compared with the negative control, whereas pretreatment with CRA-CM decreased the condensation and fragmentation of nuclei in oxidatively stressed SH-SY5Y cells (Fig. 6A).

The gene and protein expression levels of two major apoptotic signalling-related factors, Bcl-2 (B-cell lymphoma 2) and Bax (bcl-2-like protein), were measured to investigate the protective effect of CRA-CM on SH-SY5Y cells oxidatively stressed with H_2_O_2_ (200 μM). The *Bax/Bcl-2* ratio of mRNA expression increased 2.64-fold under H_2_O_2_ treatment (200 μM) compared with the negative control. However, treatment with CRA-CM (10^6^, 10^7^ and 10^8^ CFU/mL) significantly decreased the *Bax/Bcl-2* ratio to 47.0%, 57.1% and 54.9% of the ratio in the H_2_O_2_-only group, respectively (Fig. 6B). One-way ANOVA analysis showed a significant variation of heat-killed *R*. *albus* (*p* = 0.006, *F*-value = 6.9, *df*_1_ = 4, *df*_2_ = 10). The levels of the pro-apoptotic protein Bax decreased in oxidatively stressed cells with CRA-CM treatment (Fig. 6C). Treatment with H_2_O_2_ (200 μM) significantly increased Bax protein approximately 3.27-fold compared with the negative control (Fig. 6D). Co-treatment with CRA-CM (10^6^, 10^7^ and 10^8^ CFU/mL) significantly decreased the protein level of Bax to 47.4%, 48.6% and 40.4% of the ratio in the H_2_O_2_-only group, respectively. One-way ANOVA analysis showed a significant variation of heat-killed *R*. *albus* (*p* = 0.01, *F*-value = 5.9, *df*_1_ = 4, *df*_2_ = 10).

### Effect of heat-killed *R*. *albus* on body weight, hepatotoxicity and kidney toxicity

The effects of heat-killed *R*. *albus* on body weight change and serum proteins were measured in animals subjected to sodium arsenate-induced oxidative stress. None of the groups showed a significant difference in body weight changes (Supplementary Table S1). To evaluate the hepatotoxicity and kidney toxicity of heat-killed *R*. *albus*, serum glutamic oxaloacetic transaminase (AST), glutamic pyruvic transaminase (ALT), creatinine (CRE) and blood urea nitrogen (BUN) were measured. Serum ALT and BUN increased in the arsenate-treated group compared with the negative control. However, the group treated with heat-killed *R*. *albus* did not show a significant increase in any serum test, indicating that the treatment with heat-killed *R*. *albus* did not show hepatotoxicity or kidney toxicity.

### Effects of heat-killed *R*. *albus* on ROS levels in the rat brain

**Figure 7:**
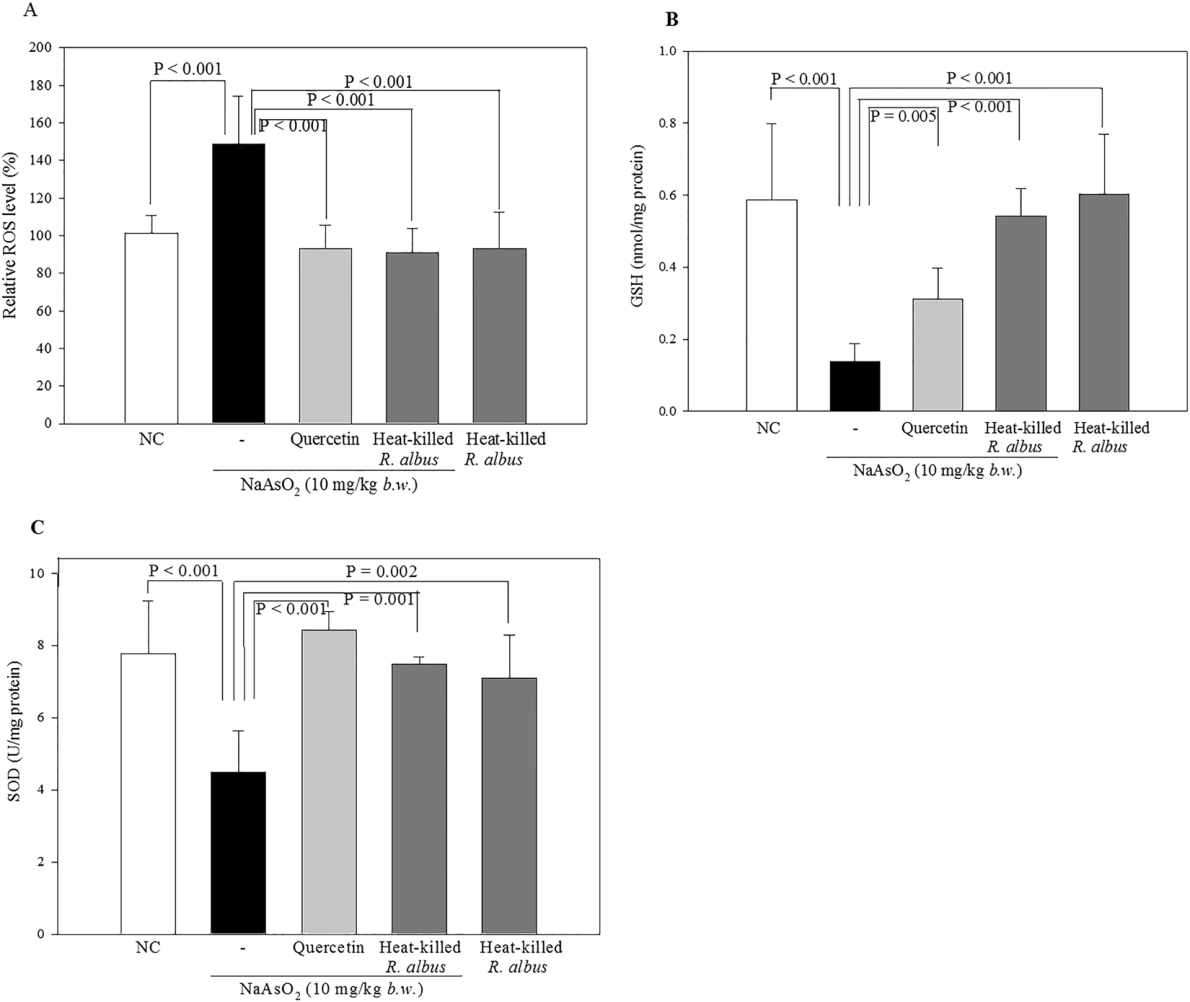
Effect of heat-killed *R. albus* on ROS levels and antioxidant activity in brain homogenates of oxidatively stressed rats. Sodium arsenate (10 mg/kg) was used to induce oxidative stress in the rat brain. The ROS levels were evaluated using the dichlorofluorescein diacetate (DCF-DA) method (**A**). GSH levels of brain tissues in rats (**B**). SOD activity levels of brain tissue in rats (**C**). The data are expressed as the mean ± SD.

Increased levels of ROS may cause oxidative stress resulting in damage to cells and tissues. Quercetin, a well-known antioxidant, was used as an experimental control. Treatment with sodium arsenate (10 mg/kg) significantly increased the ROS level in rat brain homogenate (148.61 ± 25.76%) compared with the normal control (100%) (Fig. 7A). The ROS level in brain homogenate of rats treated with heat-killed *R*. *albus* alone was 92.94 ± 19.44% compared with the normal control (100%). The ROS levels of rat brain homogenates did not increase in oxidatively stressed rats treated with quercetin (10 mg/kg) or with heat-killed *R*. *albus* (10^9^ CFU). The ROS levels were 93.10 ± 12.43% and 91.00 ± 13.06% in oxidatively stressed rats treated with quercetin and heat-killed *R*. *albus*, respectively, compared with the normal control, which indicated that ROS levels in brain homogenate of oxidatively stressed rats was reduced by heat-killed *R*. *albus* and reached a level comparable to those of normal rats and quercetin-treated rats. One-way ANOVA analysis showed a significant variation of heat-killed *R*. *albus* (*p* < 0.001, *F*-value = 12.4, *df*_1_ = 4, *df*_2_ = 25).

### Effects of heat-killed *R*. *albus* on antioxidant activity in the oxidatively stressed brain

Oxidative stress induced by arsenate significantly decreased the glutathione (GSH) level (0.14 ± 0.05 nmol/mg protein) in the rat brain tissue homogenates compared with the normal control (0.59 ± 0.21 nmol/mg protein), whereas treatment with quercetin (10 mg/kg) or heat-killed *R*. *albus* (10^9^ CFU) prior to arsenate exposure increased GSH levels to 0.31 ± 0.08 nmol/mg protein and 0.54 ± 0.08 nmol/mg protein, respectively (Fig. 7B). The GSH level in the heat-killed *R*. *albus*-only group was 0.60 ± 0.17 nmol/mg protein. Although heat-killed *R*. *albus* (10^9^ CFU) did not induce a significant increase in GSH compared with the normal control, it significantly prevented the decrease in GSH level by oxidative stress. Heat-killed *R*. *albus* (10^9^ CFU) prevented the GSH decrease more strongly than quercetin (10 mg/kg). One-way ANOVA analysis showed a significant variation of heat-killed *R*. *albus* (*p* < 0.001, *F*-value = 14.1, *df*_1_ = 4, *df*_2_ = 25).

Exposure to arsenate significantly reduced the activity of superoxide dismutase (SOD) (4.48 ± 1.17 U/mg protein) in the brain homogenates compared with the normal control (7.77 ± 1.47 U/mg protein) (Fig. 7C). Treatment with quercetin (10 mg/kg) or heat-killed *R*. *albus* (10^9^ CFU) prior to arsenate exposure increased SOD activity to 8.42 ± 0.52 U/mg protein and 7.48 ± 0.20 U/mg protein, respectively. The SOD activity of the heat-killed *R*. *albus*-only group showed 7.09 ± 1.20 U/mg protein. One-way ANOVA analysis showed a significant variation of heat-killed *R*. *albus* (*p* < 0.001, *F*-value = 10.9, *df*_1_ = 4, *df*_2_ = 20). The results showed that heat-killed *R*. *albus* restored antioxidant activity in the oxidatively stressed rat brain.

### Histological analysis of brain, liver and kidneys

**Figure 8:**
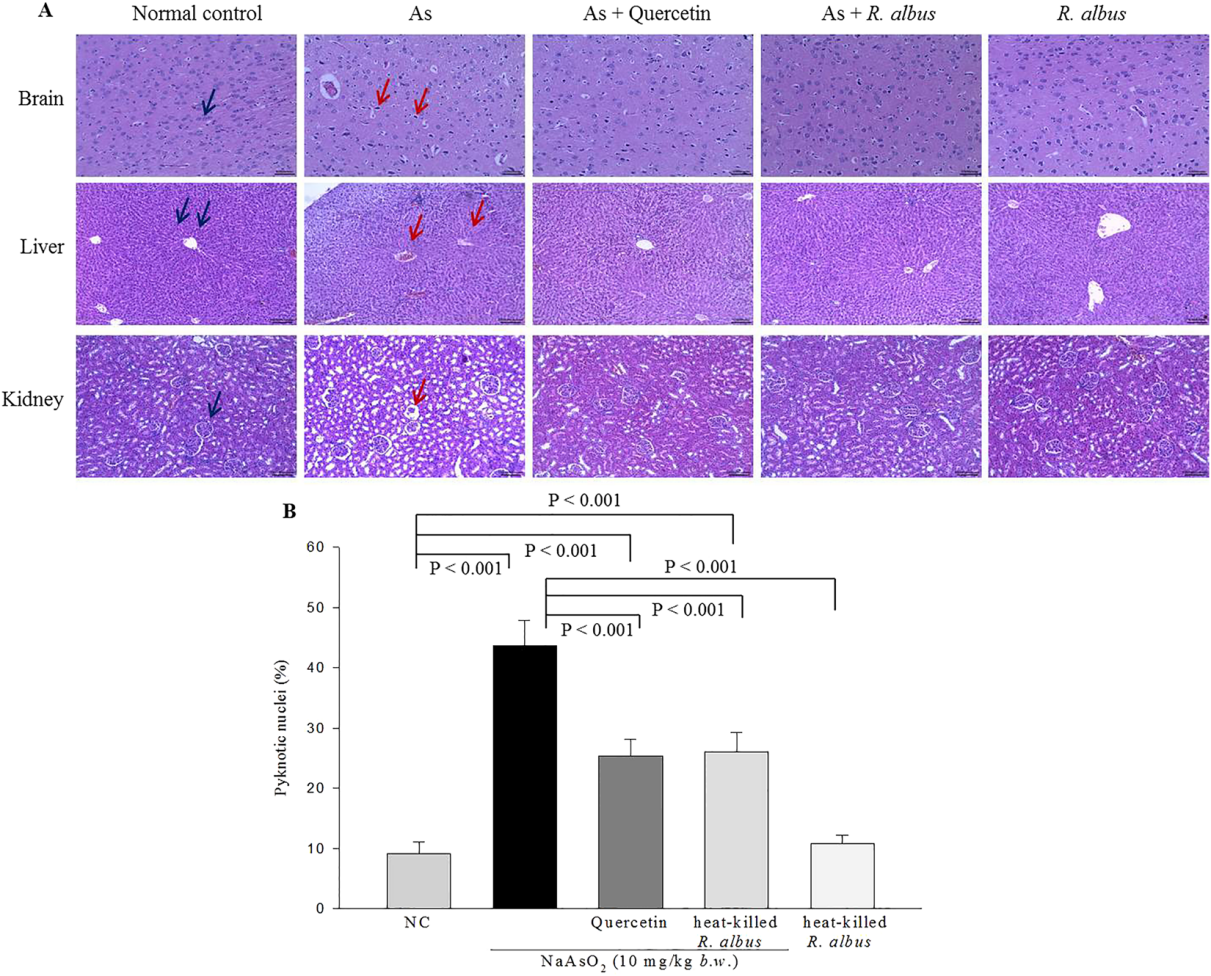
Histological analysis of brain, liver, and kidneys of rats oxidatively stressed with sodium arsenate (As). (**A**) All images were captured at 100× magnification except the brain images (200×). The blue and red arrows show normal and damaged tissues, respectively. (**B**) The percent of pyknotic nuclei per field (140 μm^2^) in brain (n = 5).

To evaluate the organ cytotoxicity of heat-killed *R*. *albus*, histopathological analysis was conducted on brain, liver and kidney tissues from the experimental rats. Administration of arsenate caused the tissues to become disorganized (Fig. 8). Normal brains showed normal nuclei that had dispersed chromatin and prominent nucleoli (blue arrow), whereas the brains of the arsenic acid-treated group showed pyknotic, darkly stained nuclei (red arrow) (Fig. 8A). Although pyknotic, darkly stained nuclei were also shown in rat brains treated with quercetin or heat-killed *R*. *albus* prior to treatment with arsenate, the percent of pyknotic, darkly stained nuclei was lower in those brains than in brains treated with arsenate alone (Fig. 8B). One-way ANOVA analysis showed a significant variation of heat-killed *R*. *albus* (*p* < 0.001, *F*-value = 38.5, *df*_1_ = 4, *df*_2_ = 20). Upon histological examination of the liver, the tissue of normal livers showed hepatic lobules and hepatocytes arranged in cords radiating from the central canal (blue arrow). Histological assessments of the liver of arsenate-treated rats revealed focal apoptosis of hepatocytes with a disrupted portal vein and hepatic necrosis (red arrow) compared with the normal liver (Fig. 8A). Histological examination of the renal tissues of the arsenate-treated rats revealed glomerular damage compared with the normal control. Shrunken renal corpuscles and intra-tubular casts (red arrow) were found in the kidneys of the arsenate-treated rats (Fig. 8A). The tissues of the rats pretreated with quercetin or heat-killed *R*. *albus* appeared nearly normal upon histological examination, indicating that heat-killed *R*. *albus* might counteract the tissue damage induced by oxidative stress.

## Discussion

Numerous studies have indicated that the gut microbiota has an important role in gut-brain crosstalk and is also linked to neuropsychological disorders. The bidirectional neuronal signalling pathways involved in this crosstalk remain poorly understood despite their important roles. The interaction between the gastrointestinal (GI) microbiota and intestinal epithelial cells plays an important role in the development of mucosal and systemic immunity as well as in the prevention and treatment of inflammatory bowel disease, periodontal disease, rheumatoid arthritis, atherosclerosis, allergy, multiorgan failure and colon cancer^24^. In addition to lactic acid bacteria and other probiotics, the gut microbiota has been studied as a beneficial influence on many aspects of human health. In this study, heat-killed bacteria were used and meaningful effects of heat-killed bacteria on neuroprotective effect were obtained *in vitro* and *in vivo* study. We assume that cell component(s) (e.g., cell wall polysaccharide and membrane proteins etc.) may be involved in those effects. Thus, further study is needed to identify the active component(s) for neuroprotective action of heat-killed *R*. *albus*.

The gut microbiota-brain axis may include gut microbiota and their metabolic products. The basic premise of this study is that a bacteria existing abundantly in the intestines of healthy individuals might protect brain cells damaged by oxidative stress. In this study, we focused on the effect of heat-killed *R*. *albus* on oxidatively stressed animals. To test this hypothesis, heat-killed *R*. *albus* was administered to Caco-2 cells, a line of human gut epithelial cells, which conditioned the medium with their secretions. The resulting conditioned medium (CRA-CM) was harvested. CRA-CM increased the expression of SRF in human neuroblastoma SH-SY5Y cells, an established model for human brain cells. This response suggests that CRA-CM might exhibit a neuroprotective effect. SRF is one of the best understood DNA-binding proteins in the human proteome^25^, and its classical pathway involves growth factor stimulation, mitogen-activated protein kinase signalling (MAPK), activation of growth-related genes and apoptosis. SRF-mediated transcription is also critical for neuroprotection^26^. SRF regulates the activity of many immediate-early proteins including c-Fos^27^. c-Fos is a type of AP-1 (activator protein-1), and AP-1 is known as a regulator of cellular proliferation and death^28^. Cyclins are important proteins in cell cycle regulation, and induced cyclins form complexes with specific cyclin-dependent kinases (CDKs). CDKs phosphorylate target proteins that are required for cell cycle progression. CDK2, a member of a family of serine/threonine protein kinases, participates in cell cycle regulation. c-Fos increases CDK expression, which increases cell proliferation^28^. Co-culture with CRA-CM significantly increased the protein expression levels of c-Fos and CDK2 in SH-SY5Y cells. Cell cycle progression from G1 to S is positively regulated by CDK2, 4 and 6^29,30^, which suggests that the up-regulated protein expression of CDK2 in CRA-CM-treated SH-SY5Y cells might influence the progression of the cell cycle, resulting in increased cell proliferation. Any activator factor(s) in CRA-CM might stimulate SRF that regulates transcription of c-Fos and consequently induces CDK activation. Thus, the results suggest that CRA-CM increases neuronal proliferation. In addition, mRNA expression of *BDNF* increased in Caco-2 cells treated with heat-killed *R*. *albus* and BDNF protein levels increased in CRA-CM, which estimated to play a role in stimulating the proliferation of SH-SY5Y cells. In further studies, the neuroprotective factor(s) in CRA-CM should be identified.

Two-dimensional gel analysis showed that β-tubulin was highly expressed in CRA-CM-treated SH-SY5Y cells. Microtubules, which consist of α-tubulin and β-tubulin, are involved in cell movement, intracellular trafficking and mitosis. The network of microtubules acts as a regulator that is associated with cell growth and key signalling processes^31^. SRF binds to the promoter sites of genes encoding cytoskeletal proteins^32^; thus, an increased protein expression level of SRF might be the cause of the increased β-tubulin expression. Consequently, CRA-CM might increase β-tubulin expression through SRF stimulation.

Neurons are sensitive to oxidative damage, and MPP^+^, H_2_O_2_ and sodium arsenate have been used to induce oxidative stress in neuron cells^33–35^. CRA-CM showed a significant protective effect in oxidatively stressed neurons. Bax and Bcl-2 are commonly known as apoptosis-related and anti-apoptosis-related proteins, respectively. The Bax/Bcl-2 ratio is more important than the expression of each gene individually in determining sensitivity to apoptotic stimuli^36,37^. The Bax/Bcl-2 ratio increased significantly in SH-SY5Y cells co-cultured with an oxidative stress inducer (H_2_O_2_), causing significant apoptosis. However, pretreatment with CRA-CM significantly decreased the Bax/Bcl-2 ratio and Bax protein expression, indicating that CRA-CM exhibits a neuroprotective effect in oxidatively stressed SH-SY5Y cells.

The issue of safety is very important in the administration of microbial agents. Arsenic acid is a naturally occurring compound that has been recognized as a toxicant. Arsenic acid has been used to induce oxidative stress in the brain in multiple *in vivo* animal studies^38,39^. Long-term exposure to arsenic acid can cause skin, liver, lung and bladder cancer as well as inflammation, vascular diseases and diabetes^40,41^. Arsenic acid treatment increases serum AST and ALT levels, which are both indicators of hepatic damage^42,43^. In particular, serum AST significantly increases at doses of arsenic acid ≥ 20 mg/kg/day^44^. In this study, serum AST levels did not increase in groups treated with arsenate at 10 mg/kg/day, while ALT levels significantly increased in arsenate-only treated rats. Arsenate exposure is associated with adverse kidney disease outcomes^45^, and BUN is used to evaluate kidney function. Administration of heat-killed *R*. *albus* (10^9^ CFU/day) did not appear to cause any hepatotoxicity or nephrotoxicity. In addition, heat-killed *R*. *albus* attenuates toxicity induced by administration of arsenate. Histological analysis of the liver, kidneys and brain also showed that arsenate caused damage to these organs and that pretreatment with quercetin or heat-killed *R*. *albus* mitigated the damage almost completely. Thus, administration of heat-killed *R*. *albus* (10^9^ CFU/day) in oxidatively stressed rats shows a tissue-protective effect without any toxicity in arsenate-treated rats.

GSH plays an important role in reducing oxidative stress. SOD is an antioxidant enzyme that reduces superoxide radicals. Arsenate-treated rat brain homogenates showed significantly increased ROS levels compared with normal controls, whereas SOD activity and GSH levels were significantly decreased. Pretreatment with quercetin or heat-killed *R*. *albus* significantly decreased ROS levels and did not produce any apparent decrease in SOD activity or GSH levels in arsenate-treated brain homogenates compared with those of the normal group, which means that pretreatment with quercetin or heat-killed *R*. *albus* protects rat brains from arsenate-induced oxidative stress. Heat-killed *R*. *albus* itself did not show antioxidant activity; however, its attenuating effect against oxidative stress seems similar to that of quercetin. In this study, although the active compound(s) in heat-killed *R*. *albus* did not be identified, based on genes and proteins expression, we predicted the potential mechanism involved in neuroprotection from oxidative damage by heat-killed *R*. *albus*. Oral administration of heat-killed *R*. *albus* might increase SOD, which reduces superoxide induced by oxidative stress. As superoxide production decreases, ROS level decreases and GSH level increases, consequently, resulted in the decrease of apoptosis. Thus, heat-killed *R*. *albus* inhibit oxidative stress-mediated neuron cell damage, leading to neuroprotection. In the further study, active compound(s) for neuroprotection from heat-killed *R*. *albus* is needed to identify to elucidate relevant neuroprotective mechanism(s) by heat-killed *R*. *albus*.

In conclusion, CRA-CM prepared with the gut bacterium *R*. *albus* increases neuronal proliferation and shows a protective effect in oxidatively stressed neurons. In an *in vivo* study, administration of heat-killed *R*. *albus* attenuated brain oxidative stress without any toxicity in the liver or kidneys. Therefore, *R*. *albus* could be a source of potential therapeutic approaches to alleviate oxidative stress-induced brain damage.

## Methods

### Materials

Dulbecco’s Modified Eagle Medium (DMEM), fetal bovine serum (FBS), Eagle’s minimum essential medium (MEM), fetal bovine serum (FBS) and penicillin/streptomycin for the cultivation of cells were obtained from HyClone (Logan, UT, USA). MEM nonessential amino acids and sodium pyruvate were obtained from Thermo Fisher Scientific (Waltham, MA, USA). MTT (3-[4,5-dimethylthiazol-2-yl]-2,5-diphenyltetrazolium bromide) was purchased from Ameresco (Solon, OH, USA). Lactate dehydrogenase (LDH) EZ-LDH kit was obtained from Daeil Lab Service (Seoul, Korea). Hydrogen peroxide (H_2_O_2_) and Hoechst 33258 were purchased from Molecular Probes (Eugene, OR, USA). MPP^+^, sodium arsenate, 2′,7′-dichlorofluorescein diacetate (DCF-DA) and quercetin were obtained from Sigma (St. Louis, MO, USA).

### Cell culture

Human SH-SY5Y neuroblastoma cells purchased from the Korean Cell Line Bank (Seoul, Korea) were grown in Dulbecco’s modified Eagle’s medium (DMEM) with high glucose. The cells were maintained at 37 °C with 5% CO_2_ and 95% air. Human Caco-2 epithelial colorectal adenocarcinoma cells purchased from the Korean Cell Line Bank were maintained in minimum essential medium (MEM) supplemented with nonessential amino acids and sodium pyruvate (1 mM). All media were supplemented with 10% foetal bovine serum (FBS) and 100 U/mL each of penicillin and streptomycin. Caco-2 cells from passages 34 to 50 were used because this range is known as the optimum for experimental purposes^46^. For SH-SY5Y cells, those passaged less than 30 times were used in all experiments.

### Preparation of heat-killed *R*. *albus*

*Ruminococcus albus* KCTC 15045 was obtained from the Korean Collection Type Culture (KCTC) and grown in a modified DSMZ 453 medium (Braunschweig, Germany). In this medium, *R*. *albus* was maintained under CO_2_ gas at 37 °C. Bacteria were harvested by centrifugation at 3,000 × g for 5 min and washed in phosphate-buffered saline (PBS). Then, the harvested bacteria were heat killed at 100 °C for 10 min and stored at −80 °C until use.

### Preparation of conditioned medium from Caco-2 cells treated with heat-killed *R*. *albus* (CRA-CM)

Treatment of Caco-2 cells with heat-killed *R*. *albus* was performed as previously described with some modification^47^. Caco-2 cells were plated in 6-well plates at a density of 5 × 10^5^ cells/mL and incubated for 10 days to form a confluent monolayer. After 10 days, cells were treated with different concentrations of heat-killed *R*. *albus* (1 × 10^6^, 1 × 10^7^ and 1 × 10^8^ CFU/mL) or PBS (for control) for 24 h. After 24 h, the supernatant was collected by centrifugation at 14,000 × g for 5 min followed by filtration using a 0.45 μm pore size filter. This supernatant was designated CRA-CM and was stored at −80 °C until use. In all experiments, the control was prepared from Caco-2 cells treated with PBS.

### Cytotoxicity of CRA-CM

Cytotoxicity was measured according to the enzymatic activity of lactate dehydrogenase (LDH) in the culture supernatant. SH-SY5Y cells were seeded in 96-well plates at a density of 5 × 10^4^ cells/well and incubated for 24 h. Then, the cells were treated with CRA-CM. After 24 h, cytotoxicity was determined using the EZ-LDH cell cytotoxicity assay kit. The LDH release assay was conducted according to the manufacturer’s instructions. Cytotoxicity was calculated using the following equation: % LDH release = (LDH in culture medium/total LDH) × 100. To determine the effect of CRA-CM on SH-SY5Y cell viability, an MTT assay was used. SH-SY5Y cells were plated in 96-well plates at a density of 1 × 10^5^ cells/well. Then, CRA-CM was applied for 24 h. After 24 h, MTT dissolved in a PBS solution was added, and the plates were incubated at 37 °C with 5% CO_2_ and 95% air. The absorbance was measured at 540 nm using a microplate reader (SpectraMax 340PC^384^; Molecular Devices, Sunnyvale, CA, USA), and viability was calculated as follows: cell viability (%) = [absorbance (experimental group)/absorbance (negative control group)] × 100. The control group was treated with conditioned medium prepared from Caco-2 cells cultured with a PBS solution.

### Neuroprotective effect of CRA-CM on oxidatively stressed SH-SY5Y cells

Oxidative stress was induced using H_2_O_2_, MPP^+^ and NaAsO_2_ to determine the protective effect of CRA-CM on SH-SY5Y cells. Cells (1 × 10^5^ cells/well) were cultured in 96-well plates with H_2_O_2_ (200 μM), MPP^+^ (1 mM) or NaAsO_2_ (20 μM) for 20 h after pretreatment with CRA-CM for 4 h. Then, the media were removed, and the cells were incubated with 100 μL MTT for 1 h. Absorbance was measured at 540 nm using a microplate reader. The relative cell viability (%) was calculated as the percentage relative to the negative control.

### Quantitative real-time polymerase chain reaction (qPCR)

SH-SY5Y cells were seeded in 6-well plates at a density of 1 × 10^6^ cells/mL. After 24 h, CRA-CM was applied. To evaluate the neuroprotective effects of CRA-CM, hydrogen peroxide (H_2_O_2_) was applied for 4 h after treatment with the conditioned medium. After 24 h, total RNA was extracted according to the manufacturer’s protocol using TRIzol Reagent (Bioneer, Daejon, Korea). Then, cDNA was synthesized using RevertAid First Strand cDNA Synthesis kit according to manufacturer’s instructions (Thermo Scientific, Waltham, MA, USA). PCR primers were purchased from Bioneer (Seoul, Korea) (Supplementary Table S2). The reaction was preheated to 95 °C for 10 min followed by 40 cycles at 95 °C for 20 s, 60 °C for 20 s and 72 °C for 30 s. Relative gene expression was quantified based on equal amounts of RNA (5 μg) and the average threshold (Ct value) for each gene. Glyceraldehyde-3-phosphate dehydrogenase (*GAPDH*) was used as the internal control gene^48^.

### BDNF detection

Quantification of BDNF in CRA-CM was performed using human BDNF ELISA kit (RAB0026, Sigma) according to the manufacturer’s protocol. CRA-CM (100 μL) was added into each well of the 96-well ELISA plate and incubated for 2.5 h at room temperature and then thoroughly washed to remove any unbound enzyme-labeled antibody. Subsequently, biotinylated detection antibody (100 μL) was added and incubated for 1 h at room temperature with gentle shaking and washed four times. After washing, ELISA colorimetric 3, 3′, 5, 5′-tetramethylbenzidine (TMB) reagent (100 μL) was added and incubated for 30 min at room temperature in the dark with gentle shaking. To terminate the reaction, stop solution (50 μL) was added and the absorbance was measured at 450 nm. BDNF concentration in each sample was calculated from the standard linear regression equation. Negative control was CRA-CM prepared from Caco-2 cells treated with PBS.

### Western blot analysis

Cells were seeded in culture plates at a density of 1 × 10^6^ cells/mL. After 24 h of treatment, the cells were harvested and washed with PBS. Then, the cells were resuspended in 100–200 μL of PRO-PREP^TM^ protein extraction solution (Intron, Seoul, Korea), then incubated at −20 °C for at least 20 min and centrifuged at 13,000 × g for 5 min at 4 °C. The proteins in the upper layer were collected and stored at −20 °C until use. The protein concentration was determined by a Bradford assay. An equal amount (20 µg) of protein in each sample was separated by 9.4% sodium dodecyl sulfate-polyacrylamide gel electrophoresis (SDS-PAGE). The separated proteins were transferred to a polyvinylidene difluoride membrane (Millipore, Bedford, MA, USA) using a Trans-Blot semi-dry transfer cell (Bio-Rad, Hercules, CA, USA). The membrane was incubated with 5% (w/v) skim milk in PBS (pH 7.4) containing 0.05% Tween-20 (PBS-T) at room temperature for 1 h, washed with PBS-T and then incubated with primary antibodies GAPDH (MA5-15738, endogenous control; 1:5,000 dilution, Thermo Fisher Scientific, Rockford, IL, USA), CDK2 (NB100-81842, 1:750 dilution, Novus Biologicals, Littleton, CO, USA) and Bax (633601, 1:500 dilution, Biolegend, San Diego, CA, USA) for 1 h at room temperature and against SRF (NBp1-33063, 1:500 dilution, Novus Biologicals) and c-Fos (AB1584, 1:500, Millipore) overnight at 4 °C with 5% (w/v) non-fat skim milk. After being washed with PBS-T, the membrane was agitated for 1 h at room temperature with peroxidase-conjugated secondary antibodies including anti-mouse antibodies for anti-GAPDH and anti-Bax (NCI1430KR, 1:10,000 dilution, Thermo Fisher Scientific), anti-rabbit antibodies for anti-CDK2 and anti-SRF (NCI1460KR, 1:10,000 dilution, Thermo Fisher Scientific) and anti-sheep antibody for anti-c-Fos (SC-2473, 1:10,000 dilution, Santa Cruz Biotechnology, Santa Cruz, CA, USA). The labeled proteins were detected with a SuperSignal West Femto Maximum Sensitivity Substrate kit (Thermo Fisher Scientific). The blots were analysed using an imaging system (FluorChem E; Proteinsimple, San Jose, CA, USA). The protein bands were quantified using analysis tools.

### Cell apoptosis assay

To observe apoptotic SH-SY5Y cells, Hoechst 33258 staining was performed as previously described^49^. Briefly, SH-SY5Y cells were plated at 2 × 10^5^ cells/well in chamber culture slides and incubated overnight. Then, the cells were pretreated with CRA-CM prior to treatment with H_2_O_2_ at a concentration of 200 μM. After 12 h, the cells were fixed for 10 min at room temperature with 4% formaldehyde diluted with PBS. Then, the cells were washed with PBS and exposed to Hoechst 33258 (5 μg/mL) for 10 min at 37 °C. To decrease background noise, the cells were rinsed with PBS. The morphological features were observed with a fluorescence microscope (Olympus Ix81-FV1000, Tokyo, Japan).

### Sodium arsenate-induced animal model of oxidative stress

Healthy Sprague Dawley rats, 6 weeks old and weighing 180–200 g, were obtained from Koatech (Gyeonggi-do, Pyeongtaek, Korea). They were housed and maintained at constant temperature (24 ± 1 °C) and humidity (55%) with alternating 12-h periods of light and dark in standard housing cages in a specific-pathogen-free environment. All animals had free access to water and were provided a normal diet (Purina Irradiated Laboratory Chow 38057, Purina Korea, Seoul, Korea). All experimental procedures were approved by the Korea University Institutional Animal Care and Use Committee (Approval No. KUIACUC-2016-131) and performed in accordance with the Guide for the Care and Use of Laboratory Animals (NIH Publication No. 85-23, 1996). After 7 days of acclimation, the animals were randomly divided into five groups (n = 8 for each), and oxidative stress was induced using NaAsO_2_ (10 mg/kg). Group I, treated with PBS (0.5 mL) alone for 15 days, served as the normal control. Group II served as a model group, receiving orally administered PBS for 15 days and NaAsO_2_ for the final 10 days. Group III served as an experimental control group, receiving orally administered quercetin (10 mg/kg) via gavage for 15 days and NaAsO_2_ for the final 10 days. Group IV received orally administered heat-killed *R*. *albus* (1 × 10^9^ CFU) via gavage for 15 days and NaAsO_2_ for the last 10 days. Group V was treated with heat-killed *R*. *albus* (1 × 10^9^ CFU) alone for 15 days.

### Body weight and serum analysis

The body weights of the animals were measured regularly throughout the experimental period. After the 15-day treatment period, the rats were anaesthetized and sacrificed. The blood was quickly collected and then was allowed to clot at room temperature for 1 h and centrifuged at 3,000 × g for 5 min to obtain serum. To evaluate the hepatotoxicity and kidney toxicity of heat-killed *R*. *albus*, AST, ALT, CRE and BUN were measured. Serum analysis was performed using a FUJI DRI-CHEM 4000i instrument and reagents (FUJIFILM Co., Tokyo, Japan).

### Measurement of ROS and GSH levels

Brain homogenates (20%, w/v) were prepared in cold 0.1 M PBS containing 1 mM EDTA (pH 7.4). The homogenates were centrifuged at 2,000 × g for 10 min at 4 °C to discard debris. Then, the homogenates were centrifuged again at 10,000 × g for 30 min at 4 °C. The supernatant was collected and used for the experiments. The protein content of the samples was measured using a Bradford protein assay with bovine serum albumin (BSA) as a standard. ROS levels in rat brain tissues were measured as previously described with some modification^50^. Brain homogenates (20 μL) were mixed with 0.1 M phosphate buffer (170 μL). Then, DCF-DA (20 μM) was added and incubated for 30 min at 37 °C. The fluorescence levels of the samples were measured using a PerkinElmer Victor plate reader (excitation 485 nm, emission 525 nm) (Waltham, MA, USA). GSH levels were measured according to the method previously described^51^ with some modification. Equal volumes of trichloroacetic acid (TCA) and homogenate were mixed and centrifuged at 2,000 × g for 10 min. Then, 20 μL of supernatant was transferred to a 96-well plate, and 5,5′-dithio-bis-[2-nitrobenzoic acid] (DTNB) was added to each well. The absorbance was measured using a SpectraMax 340PC^384^ microplate spectrophotometer (Molecular Devices) at 412 nm. The GSH contents were calculated in term of GSH/mg protein.

### Superoxide dismutase (SOD) activity

The SOD assay was performed as previously described^52^ with some modification. The homogenate (50 μL), 75 mM of Tris-HCl buffer (pH 8.2), 30 mM EDTA and 2 mM pyrogallol were mixed in a 96-well plate. The absorbance was recorded at 420 nm for 2 min using a SpectraMax 340PC^384^ microplate spectrophotometer (Molecular Devices). The activity of SOD was expressed as units/mg protein.

### Histological analysis

For histological analysis, after the 15-day treatment period, the rats were anaesthetized and sacrificed, and then the cerebrums, kidneys and livers were sectioned, fixed in 10% formalin and embedded in paraffin. Sections were cut at a thickness of 5 μm and were stained with haematoxylin and eosin (H&E). The slides were observed using an optical microscope at 100× or 200× magnification for histological changes. Pyknotic nuclei per each slide (n = 5 for each treatment group) were counted under the microscopic field (200×). Pyknotic and intact nuclei were counted per field (140 μm^2^) and total nuclei were 103 to 164 per field. The results were expressed as the percent of pyknotic nuclei per field (140 μm^2^).

### Statistical analysis

The statistical analysis was performed using one-way analysis of the variance (ANOVA) followed by Dunnett’s post hoc test using Statistical Package for the Social Science (SPSS) software package version 22.0. All data were expressed as mean ± standard deviation (SD) from at least three independent experiments performed in triplicate, and *p* value of <0.05 was considered as significant.

## Electronic supplementary material

Supplementary Information
