# Supplementary material for: Neuroprotective effect of Ruminococcus albus on oxidatively stressed SH-SY5Y cells and animals
Source: Sci Rep. 2017 Nov 6;7:14520. doi: 10.1038/s41598-017-15163-5 (PMC5674049; doi:10.1038/s41598-017-15163-5)
Supplement: Supplementary file 1 — Supplementary Information [file 41598_2017_15163_MOESM1_ESM.pdf]

Supplementary Information

Neuroprotective effect of *Ruminococcus albus* on oxidatively stressed SH-SY5Y cells and animals

Jieun Park, Jiyun Lee, Zia Yeom, Donghyuk Heo, Young-Hee Lim

Methods

2D-PAGE analysis and peptide mass fingerprinting

SH-SY5Y cells were cultured with CRA-CM treated with heat-killed *R. albus* for 24 h. Cultured cell pellets were washed twice with ice-cold PBS and sonicated for 10 sec by Sonoplus (Bandelin electronic, Germany) in 2D-PAGE sample lysis solution composed with 7 M urea, 2 M Thiourea containing 4% (w/v) 3-[(3-cholamidopropy)dimethyammonio]-1-propanesulfonate (CHAPS), 1% (w/v) dithiothreitol (DTT) and 2% (v/v) pharmalyte and 1mM benzamidine. Proteins were extracted for 1 h at room temperature by vortexing. After centrifugation at 15,000 × g for 1 h at 15°C, insoluble material was discarded and soluble fraction was used for two-dimensional gel electrophoresis. Protein concentration was assayed by Bradford method. IPG dry strips (4 - 10 NL IPG, 24 cm, Genomine, Korea) were equilibrated for 12 - 16 h with 7 M urea, 2 M thiourea containing 2% 3-[(3-cholamidopropy) dimethyammonio]-1-propanesulfonate (CHAPS), 1% dithiothreitol (DTT), 1% pharmalyte and respectively loaded with 300 μg of sample. Isoelectric focusing (IEF) was performed at 20°C using a Multiphor II electrophoresis unit and EPS 3500 XL power supply (Amersham Biosciences, Piscataway, NJ, USA) following manufacturer's instruction. For IEF, the voltage was linearly increased from 150 to 3,500 V during 3 h for sample entry followed by constant 3,500 V, with focusing complete after 96 kVh. Prior to the second dimension, strips were incubated for 10 min in equilibration buffer (50 mM Tris-Cl, pH6.8 containing 6 M urea, 2% SDS and 30% glycerol), first with 1% DTT and second with 2.5% iodoacetamide. Equilibrated strips were inserted onto SDS-PAGE gels (20 × 24 cm, 10-16%). SDS-PAGE

was performed using Hoefer DALT 2D system (Amersham Biosciences) following manufacturer's instruction. 2D gels were run at 20°C for 1,700 Vh. And then 2D gels were silver stained as described by Oakley et al[1], but fixing and sensitization step with glutaraldehyde was omitted. Quantitative analysis of digitized images was carried out using the PDQuest (version 7.0, BioRad) software according to the protocols provided by the manufacturer. Quantity of each spot was normalized by total valid spot intensity.

For protein identification by peptide mass fingerprinting, protein spots were excised, digested with trypsin (Promega, Madison, WI), mixed with α-cyano-4-hydroxycinnamic acid in 50% acetonitrile/0.1% TFA, and subjected to MALDI-TOF analysis (Microflex LRF 20, Bruker Daltonics) as described Fernandez J et al[2]. Spectra were collected from 300 shots per spectrum over m/z range 600-3000 and calibrated by two point internal calibration using Trypsin auto-digestion peaks (m/z 842.5099, 2211.1046). Peak list was generated using Flex Analysis 3.0. Threshold used for peak-picking was as follows: 500 for minimum resolution of monoisotopic mass, 5 for S/N. The search program MASCOT, developed by The Matrixscience (http://www.matrixscience.com/), was used for protein identification by peptide mass fingerprinting (PMF). The following parameters were used for the database search: trypsin as the cleaving enzyme, a maximum of one missed cleavage, iodoacetamide (Cys) as a complete modification, oxidation (Met) as a partial modification, monoisotopic masses, and a mass tolerance of ± 0.1 Da. PMF acceptance criteria is probability scoring.

References

1. Oakley, B. R., Kirsch, D. R. & Morris, N. R. A simplified ultrasensitive silver stain for detecting proteins in polyacrylamide gels. *Analytical biochemistry* **105**, 361−363 (1980).
2. Fernandez, J., Gharahdaghi, F. & Mische, S. M. Routine identification of proteins from sodium dodecyl sulfate-polyacrylamide gel electrophoresis (SDS-PAGE) gels or polyvinyl difluoride membranes using matrix assisted laser desorption/ionization-time of flight-mass spectrometry (MALDI-TOF-MS). *Electrophoresis* **19**, 1036−1045 (1998).

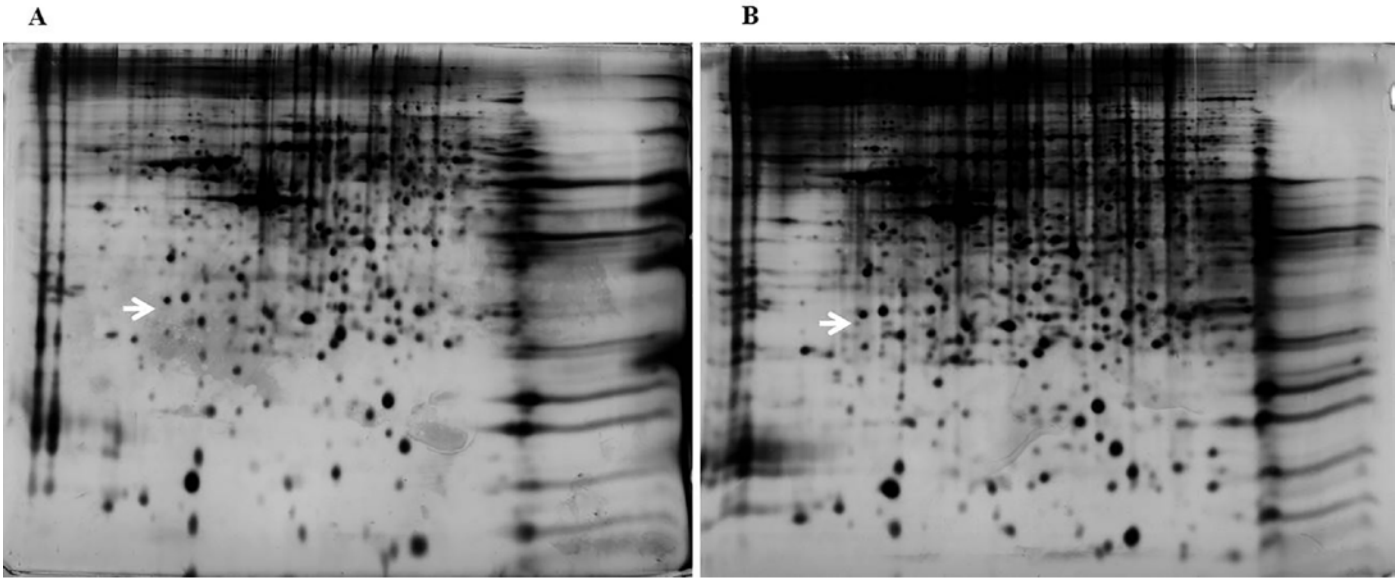

Figure S1. 2D-PAGE analysis of SH-SY5Y cells treated with CRA-CM. 2DE was performed to evaluate the effect of CRA-CM on protein expression of SH-SY5Y cells. To identify the protein, Peptide mass fingerprinting (PMF) was accessed. SH-SY5Y cells were cultured with CRA-CM treated with PBS (A) and heat-killed *R. albus* (B) for 24 h. For picking proteins, three independent experiments were performed. A white arrow is β-tubulin.

Figure S2. Western blot data. The full-length blots show GAPDH (A), SRF (B), C-Fos (C) and CDK-2 (D).

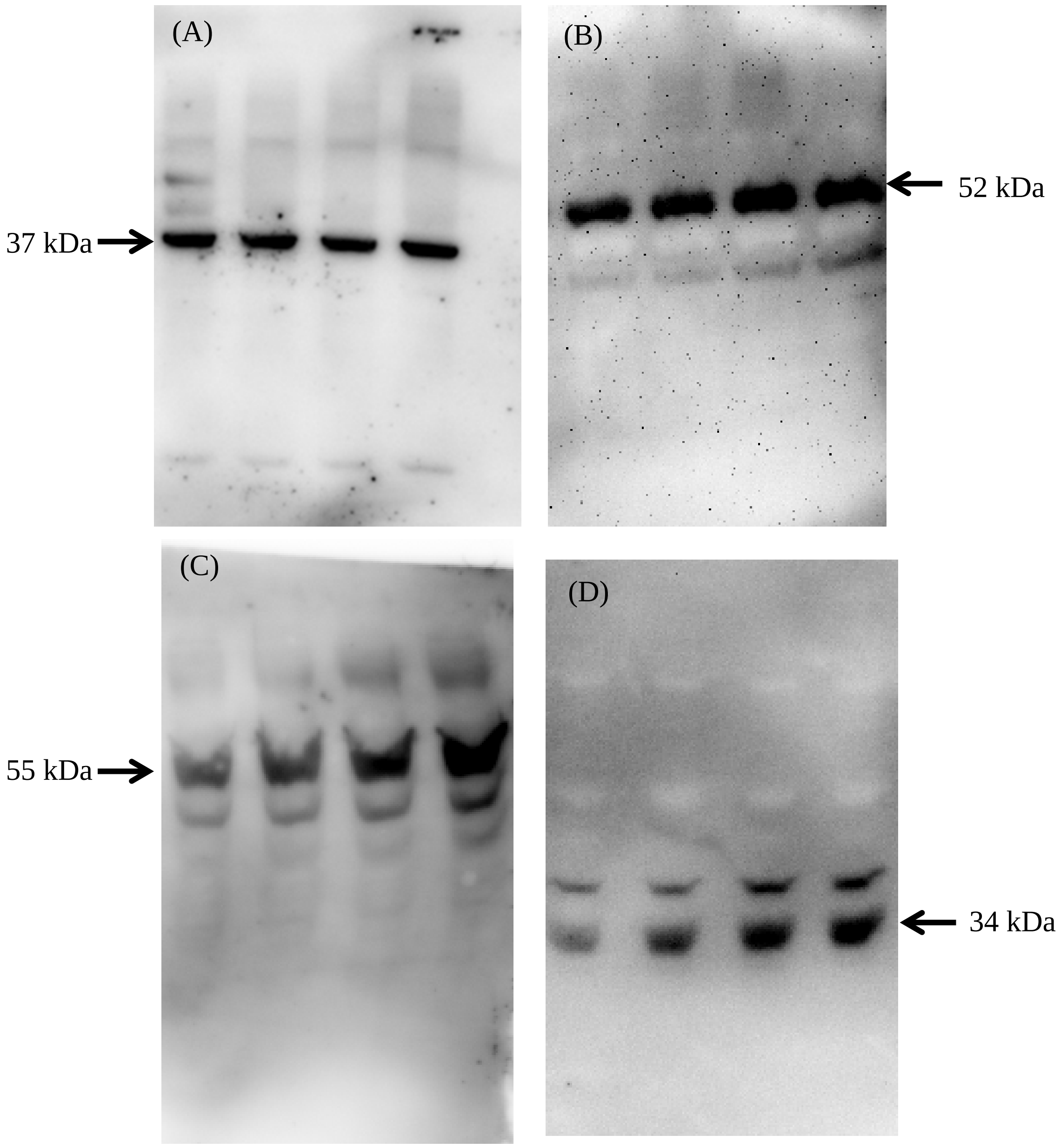

Figure S3. Western blot data. The full-length blots show β-actin (A) and β-tubulin (B).

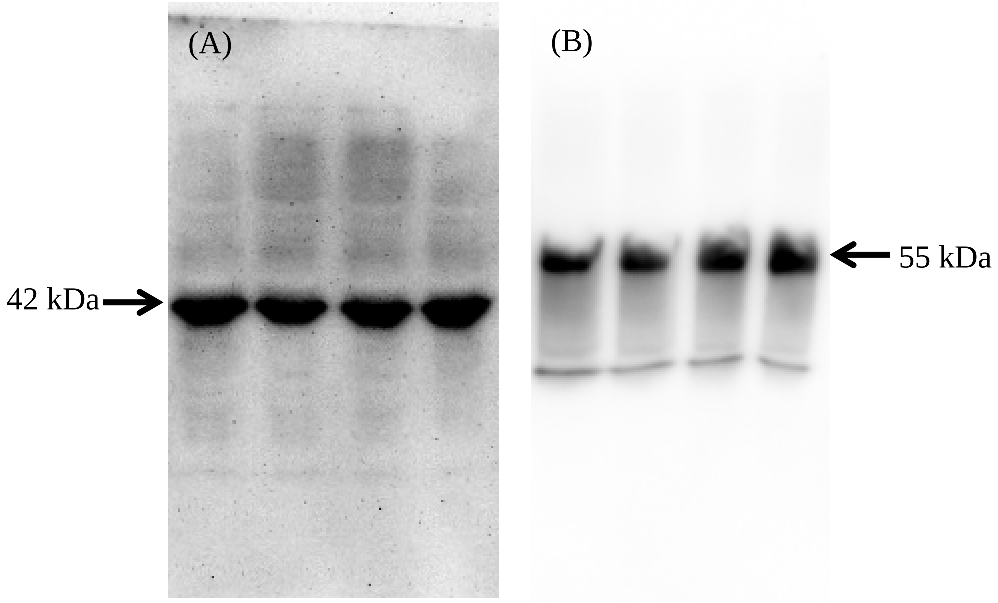

Figure S4. Western blot data. The full-length blots show β-actin (A) and Bax (B).

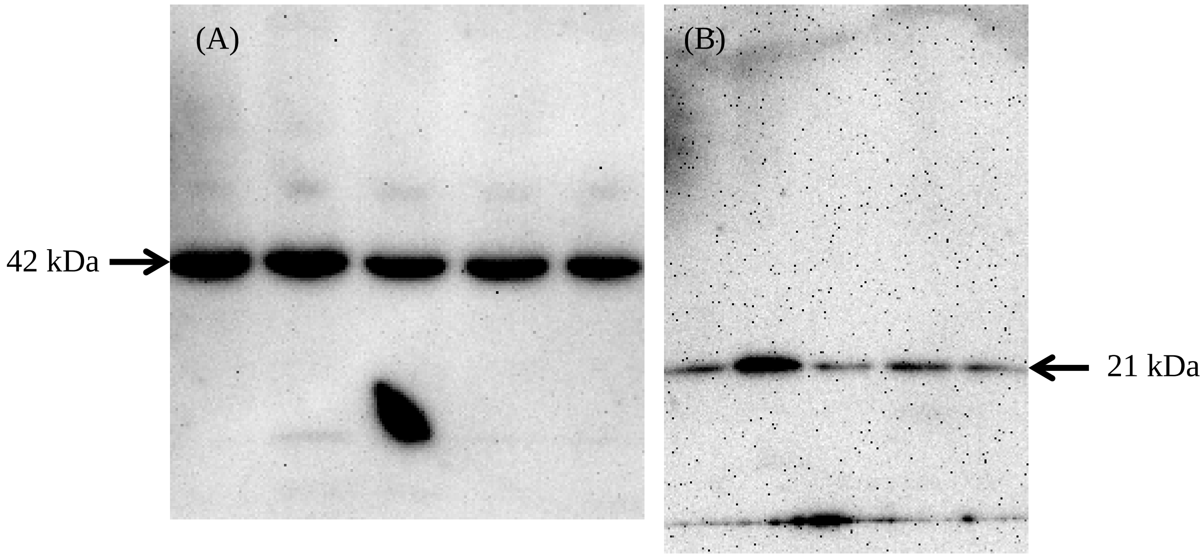

Table S1. Body weight change and serum analysis of the animals fed heat-killed *R. albus* for 14 days.

Treatment	Body weight change (g)	Serum levels			
		AST (U/l)	ALT (U/l)	CRE (mg/dL)	BUN (mg/dL)
Normal control	90.86 ± 7.42	52.25 ± 19.34	21.25 ± 4.50	0.19 ± 0.04	10.69 ± 1.85
As	97.08 ± 8.74	59.13 ± 20.35	32.50 ± 5.21[a]	0.21 ± 0.06	18.03 ± 2.52[a]
As + Quercetin (10 mg/kg)	80.83 ± 9.40	54.00 ± 15.46	20.88 ± 4.16	0.20 ± 0.05	12.13 ± 2.81
As + Heat-killed *R. albus* (10^9 CFU/mL)	97.33 ± 11.32	57.38 ± 9.32	23.50 ± 6.32	0.15 ± 0.05	11.29 ± 2.17
Heat-killed *R. albus* (10^9 CFU/mL)	80.95 ± 12.00	62.50 ± 21.49	23.13 ± 3.87	0.16 ± 0.05	12.69 ± 2.49

As, sodium arsenate

“a” indicates the significant difference compared with the normal control ($p < 0.001$).

Table S2. Primer sequences used in this study.

Gene		Primer sequence
GAPDH	Forward	5' GAGTCAACGGATTTGGTCGT 3'
	Reverse	5' GACAAGCTTCCCGTTCTCAG 3'
BDNF	Forward	5' CAAACATCCGAGGACAAGGTGG 3'
	Reverse	5' CTCATGGACATGTTTGCAGCATCT 3'
Bax	Forward	5' GTGGTTGCCCTCTTCTACTTTGC 3'
	Reverse	5' GAGGACTCCAGCCACAAAGATG 3'
Bcl-2	Forward	5' CGGCTGAAGTCTCCATTAGC 3'
	Reverse	5' CCAGGGAAGTTCTGGTGTGT 3'
